# Genome sequences of three electrochemically active bacteria isolated from an anaerobic digester

**DOI:** 10.1128/mra.01132-24

**Published:** 2024-12-10

**Authors:** Daichi Yoshizu, Keisuke Tomita, Miyu Tsuchiya, Atsushi Kouzuma, Kazuya Watanabe

**Affiliations:** 1 School of Life Sciences, Tokyo University of Pharmacy and Life Sciences, Hachioji, Tokyo, Japan; 2 J & T Recycling Corp., Tsurumi, Kanagawa, Japan; Rochester Institute of Technology, Rochester, New York, USA

**Keywords:** electroactive microbe, bioelectrochemistry, microbial electrochemical technology, microbial fuel cell

## Abstract

Three electrochemically active bacteria (EAB), strains ADMFC1, ADMFC2, and ADMFC3, were isolated from an anaerobic digester. Here, we report on genome sequences of the three EAB, which help deepen our understanding of the diversity and niches of EAB inhabiting anaerobic digesters.

## ANNOUNCEMENT

Electrochemically active bacteria (EAB) are the focus of recent research for their application in a variety of biotechnology processes, including microbial fuel cells (MFCs) (1). Attempts have been made to isolate novel EAB from the environment, while our knowledge of the diversity of EAB is still limited. Our laboratory operated MFCs filled with effluents from an anaerobic digester as the sole source of organics and microbes (located at 35.64 N, 139.39 E) (2). In that work, although these MFCs attained relatively high outputs, well-known taxa of EAB, such as *Geobacteraceae*, were not found (2). We have therefore attempted to isolate EAB from these MFCs, and a recent work isolated three EAB, namely strains ADMFC1, ADMFC2, and ADMFC3 (3). These EAB are phylogenetically diverse and affiliated with the phyla *Bacillota*, *Campylobacterota*, and *Deferribacterota*, respectively, based on partial 16S rRNA gene sequences. In addition, these EAB are considered to represent novel taxa. We were therefore interested in characterizing the genomes of these EAB.

These EAB were grown anaerobically at 30°C in DSM826 medium, in which acetate serves as the electron donor and fumarate serves as the electron acceptor (3). Genomic DNA was extracted from cultures using FavorPrep tissue genomic DNA extraction kit (Favorgen, Ping Tung, Taiwan). DNA was fragmented using a g-tube (Covaris, Woburn, MA, USA), and 10 to 20 kbp fragments were used to construct a genomic library using SMRTbell Express Template Prep kit 2.0 (PacBio, Menlo Park, CA, USA). After polymerization using Binding kit 2.2 (PacBio), it was sequenced using Sequel IIe (PacBio). SMRT Link (version 11.0.0.146107, PacBio) was used to remove adaptor sequences, and consensus sequence reads with an average quality value of less than 20 per read were removed to prepare HiFi reads. After short reads (<1,000 bp) were removed using Filtlong (version 0.2.1, https://github.com/rrwick/Filtlong), they were assembled into contigs using Flye version 2.9.1-b1780 (4) and Bandage version 0.8.1 (5), in which overlapping ends on circular contigs were removed. The completeness of the assembled genome was checked using CheckM version 1.2.2 (6), and coding sequences were predicted using DFAST version 1.2.20 (7). Default parameters were used for all software unless otherwise specified.

Genome statistics for these EAB are summarized in Table 1. The genome of ADMFC3 is not complete probably owing to the presence of repeated sequences. In addition to general features, numbers of genes encoding cytochrome *c* (cyt-c) are also presented in Table 1, since genomes of some EAB are known to encode large numbers of cyt-c, such as nearly 100 genes in *Geobacter sulfurreducens* (8). Table 1 shows that the genomes of these EAB also encode relatively large numbers of cyt-c genes. Detailed analyses of their genomes will provide us with novel insights into the diversity of bacterial extracellular electron-transport pathways.

**TABLE 1:** Genome statistics for the three EAB

Feature	ADMFC1	ADMFC2	ADMFC3
Sequencing			
Total read	30,137	32,749	27,576
Total read length (bp)	248,324,500	248,143,093	247,660,481
N50 read length (bp)	8,962	8,340	9,416
Assembly			
Scaffold	1	1	2
Level	Complete	Complete	Scaffold
Genome length (bp)	3,596,777	1,860,218	3,174,704
Coverage	69.0	59.3	78.0
Completeness (%)^*a*^	99.4	100	100
G + C content (%)	43.5	33.6	44.4
Plasmid	0	0	0
Annotation			
CDS	3,298	1,857	2,991
rRNA operon	11	3	2
tRNA gene	106	47	44
Cyt-c gene	97	85	125
Taxonomy^*b*^	*Eubacteriales*	*Sulfurospirillaceae*	*Geovibrio*
Average nucleotide identity to the closest genome (%)^*c*^	66.1 (*Desulfosporosinus acidiphilus*, NC_018068.1)	68.0 (*Sulfurospirillum barnesii,* NC_018002.1)	80.5 (*Geovibrio thiophilus*, NZ_CP035108.1)

^*a*^ Assessed by CheckM (6).

^*b*^ The lowest taxonomic rank to which an isolate is assigned based on 16S rRNA gene sequence.

^*c*^ Calculated by ANI calculator (9).

## Data Availability

Raw reads for strains ADMFC1, ADMFC2, and ADMFC3 are available in the Sequence Read Archive (SRA) database under accession numbers DRR609296, DRR609297, and DRR609298, respectively. The genome sequences were deposited in GenBank/DDBJ under accession numbers AP038755 (ADMFC1), AP038756 (ADMFC2), and BAAFZG010000001 and BAAFZG010000002 (ADMFC3).
